# Antibiotic profiling of wild-type bacilli led to the discovery of new lanthipeptide subtilin-producing *Bacillus spizizenii* strains whose 16S rDNA sequences differ from the *B. spizizenii* typing strain

**DOI:** 10.1007/s10123-022-00266-5

**Published:** 2022-07-28

**Authors:** Markus Helfrich, Karl-Dieter Entian, Torsten Stein

**Affiliations:** 1 Life Sciences, Johann Wolfgang-Goethe-University, Max v. Laue Str. 9, 60439 Frankfurt/Main, Germany; 2 Present Address: Jennewein Biotechnologie GmbH, Maarweg 32, 53619 Rheinbreitbach, Germany; 3 Chemistry & Molecular Biotechnology, Aalen University, Beethovenstraße 1, 73430 Aalen, Germany

**Keywords:** Subtilin, Lanthipeptide, Subtilosin, Surfactin, Peptide antibiotic, *Bacillus spizizenii*

## Abstract

Two dozen field-collected *Bacillus* and a dozen *Bacillus spizizenii* wild-type strains from strain collections were selected on the basis of their antagonistic properties against the Gram-positive strain *Micrococcus luteus*. Based on their genetic and antibiotic profiles, they were characterized (subtilin encoding *spaS* gene sequences, mass spectrometric, and quantitative-reversed phase liquid chromatographic analyses, as well as the presence of the lanthionine cyclase protein SpaC by western blotting), seven novel producers of the lanthipeptide subtilin. Phylogenetic analyses of the subtilin-producing wild-type strains based on their 16S rRNA sequences showed that all seven strains could be classified as *B. spizizenii*: The field-collected strains HS and N5, as well as strains DSM 618, 1087, 6395, 6405, and 8439 from the German Collection of Microorganisms and Cell Cultures. To the best of our knowledge, all *B. spizizenii* strains described so far are characterized by the fact that they can produce a lanthipeptide of the subtilin family. Both the lanthipeptide structures and the organization and sequences of the 16S rRNA-encoding genes suggest a subdivision of *B. spizizenii* into subspecies: The subtilin-producing *B. spizizenii* strains are distinctly different from the entianin-producing *B. spizizenii* typing strain TU-B-10^ T^ (DSM 15029^ T^).

**Supplementary Information:**

The online version contains supplementary material available at 10.1007/s10123-022-00266-5.

## Introduction

Gram-positive spore-forming bacteria of the genus *Bacillus* are among the most widespread bacteria worldwide; they can be found on the one hand in the soil, in fresh or seawater, as well as in the air (Ferrari et al. 1993; Moszer et al. 2002), on the other hand in the gastrointestinal tract of ruminants and humans (Hong et al. 2005; Cutting 2011). Some *B. subtilis* strains share a long history of safe use in fermented foods such as Nattō (*B. subtilis natto*, Japanese fermented soybeans) (Sun et al. 2016) and Doenjang (*B. subtilis* together with *Aspergillus oryzae*, Korean soybean paste Yue et al. 2021). For this reason, *B. subtilis* is generally classified as safe (GRAS). According to the currently accepted definition, probiotics are “live microorganisms which provide health benefits when consumed,” for example by improving or restoring the gut flora (Hill et al. 2014). Very recently, it has been shown that lanthipeptide producing commensal strains of the human gastrointestinal tract reduce vancomycin-resistant *Enterococcus faecium* (VRE) colonization and represent potential probiotic agents (Kim et al. 2018). Lanthipeptides are gene-encoded small peptides (19–38 amino acids in length) that possess the unusual bridge-forming sulfur-containing amino acids meso-lanthionine and 3-methyl-lanthionine (Freund and Jung 1992; Stein 2005; Chatterjee et al. 2005; Arnison 2013; Letzel et al. 2014).

**Fig. 1:**
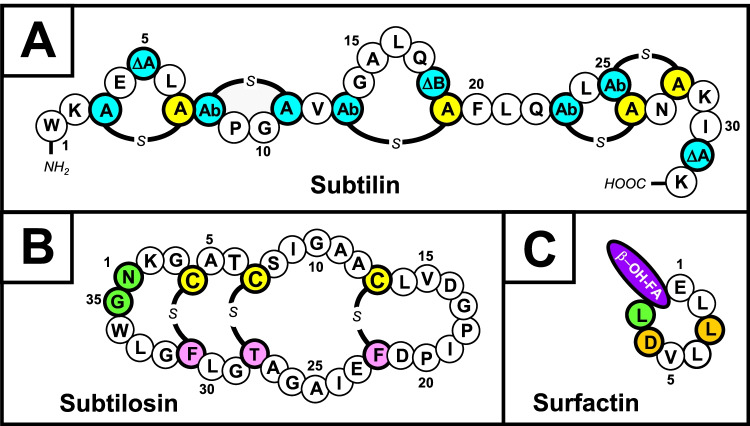
Structure of *Bacillus spizizenii* peptide antibiotics. **A** Structure of the 32 amino-acid lanthipeptide subtilin: The amino acid residues (one-letter-code) are circled and all posttranslational modified amino acid residues are colored. ΔA (blue), 2,3-didehydroalanine (dehydrated serine) at positions 5 and 32; ΔB (blue), 2,3-didehydrobutyrine (dehydrated threonine) at position 18. The five ring structures represent intramolecular thioether bridges, namely the amino acids meso-lanthionine (A-*s*-A, linking amino acids 3 and 7) or methyllanthionine (Ab-*s-*A linking amino acids 8 and 11, 13 and 19, 23 and 26, as well as 25 and 28). The posttranslational modified cysteine residue is shown in yellow. B Structure of the cyclic 35 amino-acid sactipeptide subtilosin: The amino acid residues (one-letter-code) are circled and the posttranslational modified amino acid residues are colored, namely three cross-links between the sulfurs of Cys4, 7, and 13 (yellow) which are linked to the alpha-position of Phe22, Thr28, and Phe31 (pink), respectively (Kawulka et al. 2003). The N-and C-termini (green, positions 1 and 35, respectively) are linked via amide bond forming a macrolactam. **C** Structure of the lipoheptapeptide surfactin: Surfactin is a heptapeptide lactone, which consist of a ß-hydroxy fatty acid (purple) whose carboxyl group is linked to a heptapeptide moiety via amide bond. Lactonization is accomplished by esterification of the carboxy group of the C-terminal leucine (green) with the ß-hydroxy fatty acid. Further, non-proteinogenic components are the D-configured amino acids in positions 3 and 6 (orange). Variations with both the chain length (13–15 carbon atoms) and the branching pattern (n, iso, anteiso) of the fatty acid moiety, as well as the identity of the amino acid in position 7 (exchange from L-Leu to L-Ile or L-Val) lead to a microheterogeneity of natural surfactin produced by *B. subtilis* (Kowall et al. 1998)

*B. subtilis* as well as very closely related *B. spizizenii* strains are able to produce more than two dozen antibiotics with an amazing variety of structures (Stein 2005). Peptide antibiotics represent the predominant class, among them lanthipeptides and non-ribosomal biosynthesized lipopeptide antibiotics from the surfactin, iturin, and fengycin-class (Stein 2005; Zhao et al. 2018; Caulier et al. 2019). One of the one of the earliest described lanthipeptides is subtilin, a 32-amino-acid peptide produced by a *Bacillus* strain (see Fig. 1A for a structure representation) that has been originally described 1944 (Jansen and Hirschmann 1944). The subtilin producing strain was originally isolated 1911–1912 by Karl Kellerman (Kellerman et al. 1912) and deposited as *B. subtilis* strain No. 6633 into the American Type Culture Collection (ATCC) by the “Bureau of Plant Industry, Soils, and Agricultural Engineering of the United States Department of Agriculture” (Garibaldi and Feeney 1949); this strain was proposed as *B. spizizenii* by Nakamura et al. (1999) and promoted by Dunlap et al. (2020). Furthermore, several *B. subtilis* wild-type strains produce the macrocyclic sactipeptide subtilosin A (Fig. 1B; Zheng et al. 1999; Stein et al. 2004) with a series of unusual intramolecular thioether linkages (Marx et al. 2001; Kawulka et al. 2003; Stein 2020). The lipoheptapeptide lactone surfactin (Fig. 1C) is a powerful surfactant with potent antimicrobial activities of surfactin are based on its detergent-like action on biological membranes (Heerklotz und Seelig 2001; Carrillo et al. 2003). Surfactin is distinguished by its exceptional emulsifying, foaming, antiviral, antitumor, anti-mycoplasma, and hypocholesterolemic activities (Peypoux et al. 1999; Kaspar et al. 2019).

The aim of this study was to analyze the potential of *Bacillus* strains to produce the lanthipeptide subtilin, the sactipeptide subtilosin, and the lipopeptide surfactin. Both laboratory and field collected *Bacillus* strains were taxonomically classified by gene sequencing (16SrRNA encoding *rrn* genes and subtilin encoding *spaS* gene). The antibiotic profiles were characterized under conditions optimal for the production of lanthi- and sactipeptides; MALDI mass spectrometry and reversed phase rpHPLC were used for qualitative and quantitative verification of the produced peptides. Furthermore, the presence of subtilin-modifying enzymes (SpaC, the subtilin cyclase, for example) was verified by immunoblotting.

## Materials and methods

### Bacterial strains and media

*Bacillus* strains (for a complete list see Table 1) from culture collections are from ATCC (American Type Culture Collection) and DSMZ (German Collection of Microorganisms and Cell Cultures; strain collections for microorganisms and links to them can be found in the Tab. S1, Supplement) or natural isolates (see below). For antibiotic production liquid Landy medium cultures were routinely grown aerobically at 37 °C (Landy et al. 1948) supplemented with 0.5% yeast extract (Heinzmann et al. 2006; Stein 2020). Solid media contained TY agar (0.8% tryptone, 0.5% yeast extract, 0.5% NaCl, 1.5% agar) or lysogeny broth (LB) agar (the commercial supplier of the media components was Gibco, Neu-Isenburg, Germany), standard incubation conditions were overnight (15–20 h) at 37 °C.

**Table 1:** *Bacillus* strains used in this study and their abilities to produce subtilin (Sub), subtilosin (Sbo), and surfactin (Srf)

***Bacillus* strain**	Synonyms^a^, properties	**Sub**^**b,c,d,e**^	**Sbo**^**b,c,e**^	**Srf**^**e**^	**Reference**
***Spizizenii***					
ATCC 6633	DSM 347, NRS 231 (safe), IAM 1069, Sub^+^/Sbo^+^/Srf^+^—reference strain	** + **	** + **	** + **	Garibaldi and Feeney (1946) Heinzmann et al. (2006)
DSM 618	Test strain for the detection of antibiotics in meat	** + **	** + **	** + **	This work
DSM 1087	W23	** + **	** + **	** + **	This work
DSM 6395	W23 2A2	** + **	** + **	** + **	This work
DSM 6405	mutant of W23 SR	** + **	** + **	** + **	This work
DSM 8439^ T^	W23, IAM 12,021	** + **	** + **	** + **	This work
N5	Soil isolate	** + **	** + **	** + **	This work
HS	Soil isolate	** + **	** + **	** + **	This work
TU-B-10^ T^	DSM 15029^ T^, entianin	**( +)**	** + **	** + **	Fuchs et al (2011)
***Subtilis***					
168^f^^,g^	DSM 402, ATCC 23,857 Sub^−^/Sbo^+^/Srf^−^-reference	**-**	** + **	**-**	Stein et al. (2004)
DSM 10^ T^	ATCC 6051^ T^, NCIB 3610	**-**	** + **	** + **	This work; Fuchs et al. (2011)
IP	Soil isolate	**-**	** + **	** + **	This work
DSM 2109	ATCC 11,774, NCTC 8236	**-**	** + **	** + **	This work
**Others**					
DSM 2109	ATCC 11,774, NCTC 8236	**-**	** + **	** + **	This work
DSM 3256	IAM 1213	**-**	** + **	** + **	This work
DSM 3258	IAM 1260	**-**	**-**	** + **	This work
A1/3	ericin A/S	**( +)**	**-**	** + **	(Stein et al. 2002a, b; Hofemeister et al. 2004)
HI-1	Soil isolate	**-**	**-**	** + **	This work
DSM 1088	*B. natto*	**-**	** + **	** + **	Stein et al. (2004)
DSM 2277	*B. atrophaeus* ATCC 51,189, NCTC 10,073	**-**	** + **	** + **	Fritze and Pukall (2001) Stein et al. (2004)

^a^Description and links to the strain collections are given in the Supplement (Tab. S1); ^b^PCR, gene sequencing; ^c^rp-HPLC

^d^Western blotting (SpaC/SpaB protein); ^e^MALDI-TOF MS; ^f^genome sequence (NC_000964.3; Kunst et al. 1997)

^g^*B. subtilis* 168 can be converted to a surfactin producer after point mutation of *sfp* is repaired or functional *sfp* is introduced (Lambalot et al. 1996)

### Isolation of subtilin producing Bacilli

Soil samples with different nutrient content were isolated from nutrient-rich arable land (N, 50° 11′ 1′′, N; 08° 47′ 54′′ E) and forest (IP, 50° 08′ 22′′ N; 09° 16′ 33′′ E) in Hessen, Germany, and from alpine surroundings (HI, 47° 21′ 13′′ N; 10° 06′ 01′′ E and HS, 47° 19′ 36′′ N; 10° 11′ 02′′ E; both approx. 2000 m, Vorarlberg, Austria). Twenty-five grams of soil were suspended in 100 mL of sterile water, pasteurized (10 min at 80 °C) in order to accumulate spore-forming bacteria, diluted 1: 100, plated out on TY plates and incubated at 37 °C for 15 h. To monitor the antimicrobial activities, individual colonies were replica plated on TY agar plates in Petri dishes with cams in order to obtain optimal aerobic conditions for antibiotic production (Sarstedt, Nümbrecht, Germany) with *Micrococcus luteus* ATCC 9341 as the test organism (Stein et al. 2004).

### *Molecular biology techniques*

Established protocols were followed for molecular biology techniques; *E. coli* plasmids were isolated by the rapid alkaline extraction procedure. DNA amplification using *Taq* DNA polymerase was performed according to the instruction of the commercial supplier (Boehringer GmbH; Mannheim, Germany) in a DNA Thermal cycler (Eppendorf; Hamburg, Germany). The 16S rRNA encoding genes of the bacterial isolates were PCR amplified using primers 16S_forward 5′-GAGAGTTTGATCCTGGCTCAG-3′ and 16S_reverse 5′-ACGACTTCACCCCAATCATC-3′ (Heyrman et al. 2001). The 16S rRNA sequences of the *Bacillus* strains DSM 6405, 3258, N5, HS1, IP, and HI were deposited under the NCBI GeneBank records DQ452508-13, respectively; the DSM618 sequence under record DQ529249 (for NCBI GeneBank links see Tab. S4, Supporting Informations). The structural gene of subtilin *spaS* including 250 bp of the upstream non-coding region has been PCR-amplified with primers SpaS_Seq1 (5′-CTATGAATCAATGGAAGGG-3′) and SpaS_Seq2 (5′-CTTCATTTTCTTGTCCCG-3′); GeneBank records for *B. spizizenii* DSM 618 (DQ452514: https://www.ncbi.nlm.nih.gov/nuccore/DQ452514), *B. spizizenii* DSM 6405 (DQ452515: https://www.ncbi.nlm.nih.gov/nuccore/DQ452515), HS (DQ452516: https://www.ncbi.nlm.nih.gov/nuccore/DQ452516), and N5 (DQ452517:https://www.ncbi.nlm.nih.gov/nuccore/DQ452517). Subtilosin structural gene *sbo* sequencing has been performed with primers described previously (Stein 2004); GeneBank records for novel *sbo* sequences of the natural isolates *B. subtilis* HI-1, N5, and IP are deposited under accession numbers DQ452518-20, respectively. DNA cleavage and isolation were achieved with the QIAquick™ purification kit (Qiagen GmbH; Hilden, Germany). Oligonucleotides were purchased from ARK (ARK Scientific GmbH Biosystems, Darmstadt, Germany). Sequencing was carried out by SRD (Scientific Research and Development, Oberursel, Germany); nucleotide sequences have determined at least two times for each DNA-strand.

### DNA sequence and phylogenetic analyses

The 16S rRNA sequences of *B. subtilis* strains 168 and ATCC 6051 were taken from the NCBI gene bank reference sequences NC_000964.3 and NZ_CP020102.1, respectively. The 16S rRNA sequences of the *B. spizizenii* strains ATCC 6633, W23, and TU-B-10^ T^ were taken from NZ_CP034943.1, CP002183.1, and CP002905.1, respectively. GC content calculation and GC profiling of different *B. subtilis* genomes and subtilin gene clusters was performed using ENDMEMO (http://www.endmemo.com/bio/gc.php). Multiple sequence alignment (MSA) analyses were performed with Clustal Omega 1.2.4 that uses seeded guide trees and HMM profile-profile techniques to generate alignments (Waterhouse et al. 2009; Sievers et al. 2011) (https://www.ebi.ac.uk/Tools/msa/clustalo/) or MAFFT version 7 (Katoh et al. 2019; https://mafft.cbrc.jp/alignment/server/). Phylogenetic analyses were carried out by the neighbor joining method (Saitou and Nei 1987) using MAFFT (version 7; Kuraku et al. 2013) or Clustal Omega 1.2.4 software. The NCBI BLAST server (https://blast.ncbi.nlm.nih.gov/Blast.cgi) was used for homology searches.

## MALDI-TOF mass spectrometry (MALDI-TOF MS)

Cell-free aliquots of a 500-µL culture supernatants of overnight grown *Bacillus* strains in Landy media were extracted with 500 µL 1-butanol, 400 µL of the organic phase was dried in a speed-vac evaporator, and the extracted peptides were dissolved in 5 µL 50% acetonitrile and 1% trifluoroacetic acid (v/v in H_2_O). 0.3–0.5 µL aliquots of the solutions were mixed directly on the target with 1.5 µL matrix solution, and the mixture was dried with the help of hot air. The matrix was 20 mg/mL DHBs (9:1 mixture of 2,5-dihydroxybenzoic acid and 2-hydroxy-5-methoxybenzoic acid) complemented with solubilized in aqueous solution of 50% acetonitrile and 1% trifluoroacetic acid (v/v in H_2_O). Generally, if sample spots are readily crystallized, mass spectra with sufficient signal-to-noise values were obtained from the edge of the crystals (Stein 2008). Delayed extraction™ (DE) MALDI time-of-flight (TOF) mass spectra were recorded on a Voyager-DE STR instrument (Applied Biosystems Instruments) using a nitrogen laser (λ=336 nm, repetition rate = 20 Hz) for desorption and ionization with an acquisition mass range from 600 to 15 000 m/*z* and the low mass gate set to 550 m/*z*. The total acceleration voltage was 20 kV with 68.5% grid voltage on the first grid, 0.02% guide wire voltage, 200 ns delay, and a mirror voltage ratio of 1.12. All experiments were carried out with the reflector positive ion mode. Between 500 and 1 000 laser shots were accumulated for each mass spectrum.

### Subtilin quantitation

Similar growth profiles were obtained for all *B. spizizenii* strains tested after growth in 2 mL Landy medium (37 °C, 175 rpm). In standard determinations, *B. spizizenii* was grown for 16 h, the cultures were centrifuged (4 °C, 15 min, 15,000 g), and the subtilin amount was determined in 500-µL aliquots by RP-HPLC using a Beckman Gold HPLC System and an analytical ODS Hypersil column (particle size: 5 µm, width and length: 2 × 250 mm, Maisch, Ammerbuch, Germany) as described previously (Heinzmann et al. 2006).

### SDS-PAGE and Western blotting

SDS-PAGE (10% tris–glycine gels) and Western blot analyses were performed with an immuno-purified SpaC-directed immunoserum as described previously (Helfrich et al 2007). Molecular standards were purchased from BioRad (München, Germany).

## Results and discussion

### Identification of antibiotic producer among field collected spore forming bacteria

**Fig. 2:**
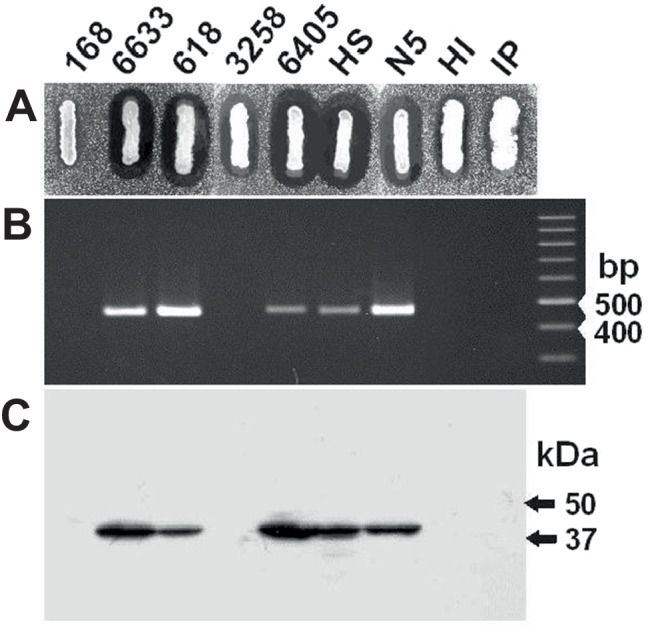
Identification of subtilin producing *Bacillus* strains. Representative examples of wild-type *Bacillus spizizenii* strains DSM 618, DSM 3258, and DSM 6405; the natural isolates are designated HS, N5, HI, and IP. The references for subtilin producers and nonproducers were the strains *B. spizizenii* ATCC 6633 (Sub^+^) and *B. subtilis* 168 (Sub^−^), respectively. **A** Analyses of *Bacillus* strains in agar diffusion tests using *M. luteus* as the test organism. The production of antimicrobial agents is indicated by growth inhibition halos of the test organism. For *B. subtilis* 168 no growth inhibition halo was detected. Large growth inhibition halos comparable to the Sub^+^ strain *B. spizizenii* 6633 were obtained for the strains HS, N5, DSM 6018, and DSM 6405. The halos for the strains DSM 3258 as well as for HI and IP were significantly smaller. B The subtilin structural gene *spaS* gene was PCR amplified using the primers SpaS_Seq1 and 2, as well as DNA from different *Bacillus* cells as template. The right lane contains a nucleotide size standard. The calculated size of the amplified DNA segment containing *spaS* is 480 bp. The subtilin gene *spaS* was identified in strains with large inhibition halos (*B. spizizenii*, HS, N5, DSM 6018, and DSM 6405), whereas it was not detected in strains with intermediate inhibition halos. **C** Western blot analyses: *Bacillus* cells harvested in the stationary growth phase were lysed. After SDS-PAGE separation of cell lysates, the proteins were transferred nitrocellulose membranes and the presence of the SpaC protein was analyzed with SpaC-directed immunoserum. Consistent with the results shown in Fig. 2B, the SpaC protein (calculated molar mass of 42,5 kDa) was found in all strains containing the subtilin gene *spaS*

We took random soil samples from various environmental habitats, nutrient-rich farmland, forest (200-m altitude, Hesse, Germany), as well as alpine environments (2000 m altitude, Vorarlberg, Austria). After pasteurization (10 min, 80 °C), spore-forming bacteria were plated on agar plates containing TY medium. Single colonies were selected and examined for their antibiotic activities against *M. luteus* as a highly sensitive Gram-positive target strain using optimized conditions for the detection of lanthipeptides (Heinzmann et al. 2006) and sactipeptides (Stein et al. 2002a, b). Twenty to 25% of the isolated aerobically grown spore-formers significantly inhibited *M. luteus* growth. Twelve potential antibiotic producers were identified: six strains from farmland (N5), one from a mixed forest (IP), and five strains from alpine environments (HS and HI). Strains HS and N5 exhibited large and clear zones of inhibition comparable to the established subtilin-producer *B. spizizenii* ATCC 6633 (Fig. 2A) and were later characterized as subtilin producers (Sub^+^, for a summary see Table 1). In contrast, strains HI and IP exhibited inhibition zones that were only about one quarter the size of those of *B. spizizenii* ATCC 6633). Both strains were classified as Sub^−^ in further studies (for a summary see Tab.1). Additionally, we tested twelve *B. subtilis* and *B. spizizenii* wild-type from the German Collection of Microorganisms (DSMZ) for their ability to inhibit *M. luteus* growth. Strains DSM 618, 1087, 6395, 6405, and 8439 showed promising antimicrobial activities with growth inhibition activities comparable to the well-characterized subtilin-producer *B. spizizenii* ATCC 6633; representative examples are given in Fig. 2A. The *B. subtilis* strains DSM 3256 and DSM 3258 exhibited semi-large inhibition zones (Fig. 2A).

### Phylogenetic classification of Bacillus strains on the basis of 16S rRNA sequences

**Fig. 3:**
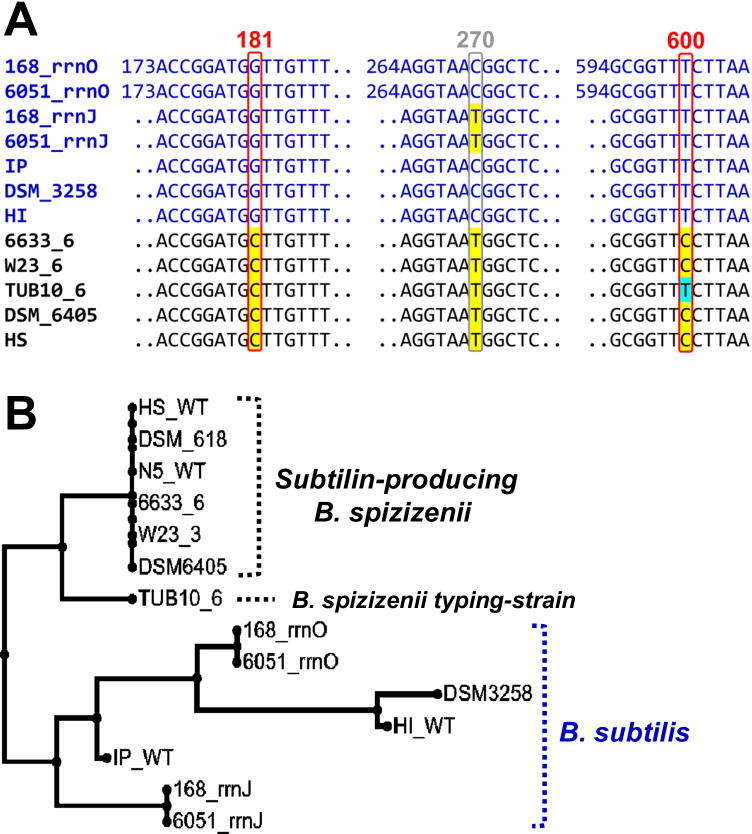
16S rRNA sequence analysis of different *Bacillus* strains. **A** Multiple sequence alignment: The sequences of the ten 16S rRNA encoding *rrn* genes of a given. *B. subtilis* strain are almost identical (99.81%; see also Tab. S4, supporting information). Two representative 16S rRNA sequences are shown for *B. subtilis* strains 168 and ATCC 6051 (*rrnO* and *rrnJ* genes). Since the 16S rRNA sequences within *B. spizizenii* strains ATCC 6633, W23, and TUB are almost identical, only one representative *rrn* sequence is shown. All 16S rRNA genes of the newly identified subtilin-producing strains exhibited 100% identity to the *B. spizizenii* reference strains ATCC 6633 and W23. The positions 181 and 600 of the 16S rRNA sequences clearly differentiated the *Bacillus* strains *B. subtilis* and *B. spizizenii* (red box). B Neighbor joining phylogenetic tree: The strains DSM 3258, HI, and IP were classified as *B. subtilis* (blue bracket) based on the similarity of their 16S rRNA sequences to those of *B. subtilis* 168 and ATCC 6051

The 16S rRNA encoding genes of all field-collected spore-forming strains were PCR amplified and sequenced. Remarkably, the 16S rRNA sequences of the field-collected *Bacillus* strains HS1 and HS2 (hereinafter referred to as strain HS) and N1, N5, and N6 (referred to as strain N5), as well as the DSMZ strains DSM 618 and DSM 6405 were identical to the database-established 16S rRNA sequences of *B. spizizenii* ATCC 6633 and W23 (Fig. 3A). This suggests their classification as *B. spizizenii*, a species first postulated by Nakamura et al. (1999) and promoted by Dunlap et al. (2020). As indicated in Fig. 3B, MSA analyses revealed that position 181 of the individual 16S rRNA encoding *rrn* genes can be used for clear species differentiation between *B. spizizenii* (C at position 181) and *B. subtilis* (G at position 181 in all ten *rrn* genes of *B. subtilis 168* and ATCC 6051).

On the other hand, the field-collected strains IP and HI1 (referred to as strain HI) as well as the laboratory strain DSM 3258 are classified as further members of *B. subtilis* (see Fig. 3B for phylogenetic analyses). 16S rDNA-based classification revealed that the field collected *Bacillus* strains HI2 and HI3 belong to *B. macroides* and *B. licheniformis*, respectively. Both species are known for members with a high potential to produce antibiotics. The strain N2 was classified as *B. thuringiensis*, a class of *Bacillus* strains some members of which produce the lipopeptide kurstakin (Hathout et al. 2000). No sequence was obtained for strain N3, and strain N4 was discarded because no further significant antibiotic activities could be identified under the test conditions used.

### Diagnostic PCR and DNA sequencing of the subtilin structural gene spaS

For PCR amplification of the subtilin structural gene *spaS* oligonucleotide primers (SpaS-Seq1 and SpaS_Seq2) complementary to the –35 region of the *spaS-*promoter and the *spaS-spaI* intergenic region were used. The presence of the *spaS* gene was verified in the case of *B. spizizenii* strains DSM 618 and 6405, as well as the natural isolates HS and N5 (Fig. 2B; for a summary see Table 1). The corresponding *spaS* genes and their flanking regions (ribosomal binding site, -10-region) were sequenced (the GeneBank records for *B. spizizenii* DSM 618, 6405, HS1, and N5 are DQ452514-17, respectively). The *spaS* sequences were identical to the *spaS* sequence of the *B. spizizenii* strain ATCC 6633 strain ATCC 6633: NZ_CP034943.1). For the *B. spizizenii* strains containing the *spaS* gene also, the subtilin cyclase SpaC (Fig. 2C) was detected within PAGE-separated cell extracts using SpaC specific immunosera. Most likely, all observed SpaC proteins of the *B. spizizenii* strains are closely related, since immunoblot signals of potential subtilin producers showed comparable intensities (Fig. 2C). In contrast, an immunoblot of EriC from *B. subtilis* A1/3, a protein exhibiting only 85% sequence identity with the SpaC counterpart from *B. spizizenii* ATCC 6633 (Stein et al. 2002a), showed only very weak immunoblotting signals. Consistently, for the strains which lack the subtilin structural gene *spaS* (strains 168, 3258, HI, and IP), no SpaC protein could be detected in the associated cell extracts either (Fig. 2B).

### Identification of subtilin within B. spizizenii culture supernatants by MALDI-TOFMS

**Fig. 4:**
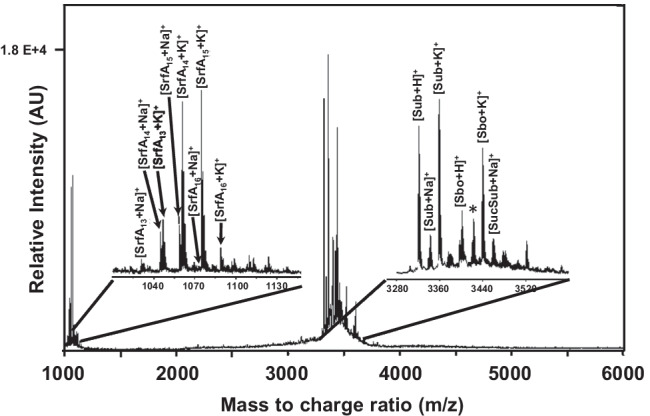
MALDI-TOF MS of a butanolic extract of a representative *B. spizizenii* (DSM 618) culture supernatant: The mass spectrum was recorded in the positive ion modus. Left insert, positively charged sodium (*m/z* 1030.7, 1044.7, 1058.7, and 1072.7) and potassium (1046.7, 1060.7, 1074.7, and 1088.7) adducts of different isoforms of the lipopeptide isoform surfactin A are labeled. The number of carbon atoms within the surfactin acyl chain is denoted in the index of SrfA (Kowall et al. 1998). In the right insert, the proton-adducts of subtilin (Sub, *m/z* 3322.8) and subtilosin (Sbo, 3400.8) are labeled as well as their sodium- and potassium adducts (Sub, 3344.8, 3360.9; Sbo, 3438.9). SucSub represents the potassium-adduct of succinylated subtilin at *m/z* 3460.9; the peak at *m/z* 3422.8 can be interpreted as [SucSub + H]^+^ or [Sbo + Na].^+^ (asterix)

MALDI-TOFMS analyses of butanolic extracts of *B. spizizenii* culture supernatants resulted in prominent peak cluster between m/z 3280 and 3520 (Fig. 4). They represent H^+^-, Na^+^-, and K^+^-adducts of the lanthipeptide subtilin and its succinylated derivative (Chan et al. 1993; Heinzmann et al. 2006), as well as the sactipeptide subtilosin, respectively (see Table 1 for summary). Furthermore, the lipoheptapeptide surfactin was identified by MALDI-MS experiments due to the characteristic *m/z* values of its different isoforms (Fig. 4). Remarkably, all investigated *B. subtilis and B. spizizenii* strains produced surfactin (see Table 1 for a summary), notably, with the exception of the laboratory-adapted *B. subtilis* strain 168. For this strain, a mutation within the 4′-phosphopantetheine transferase *sfp* gene which posttranslationally modifies the required surfactin synthetase enzymes so that surfactin cannot be produced (Lambalot et al. 1996). However, the detection of surfactin within the culture supernatants of all investigated *B. subtilis* and *B. spizizenii* strains, and the widespread frequent appearance of surfactin producers among strains of the genus *Bacillus* (Peypoux et al. 1999; Kalinovskaya et al. 2002; Torres et al. 2016) restricts the usage of the phenotype “*surfactin production*” as biomarker for subspecies classification/differentiation (e.g., between *B. subtilis* and *B. spizizenii*).

### Quantitative determination of subtilin

**Fig. 5:**
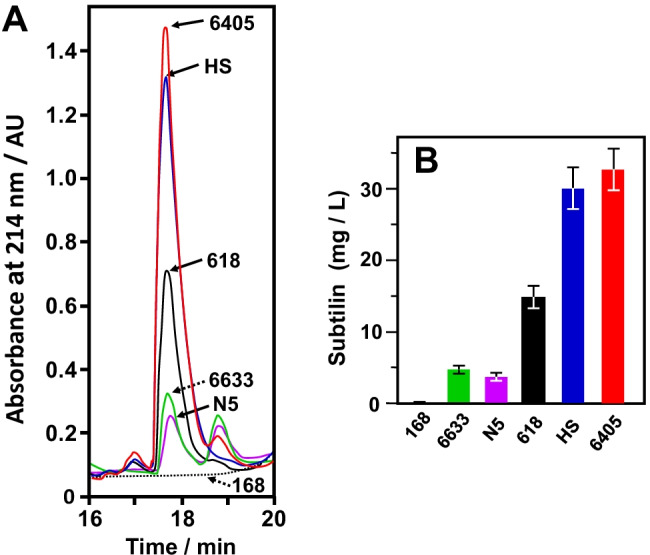
Subtilin production by different *B. spizizenii* strains. **A** Identification of subtilin by quantitative RP-HPLC. *B. spizizenii* strains were grown for 16 h in Landy medium to comparable cell densities; the strain *B. subtilis* 168 was used as a non-subtilin producing control (dotted line in black). Five hundred microliters of aliquots of cell-free culture supernatants from stationary cells were separated by reverse phase HPLC. Antimicrobial growth assays and MALDI-TOF–MS experiments indicated that subtilin elutes as a single peak at 17.5 to 18.5 min. B Quantitative determination of subtilin produced by different *B. spizizenii* strains. The integrals of the peaks eluting at 17.5–18.5 min (Fig. 6A) are proportional to the amount of subtilin. The presented values with standard errors of less than ± 12% are the means of three independent cultures for which the determinations have been performed twice

The subtilin concentration was determined in Landy-culture supernatants of stationary grown *Bacillus* cells (Fig. 5A/B). Whereas the production yields of *B. spizizenii* ATCC 6633 (4.9 mg/mL) and the field-collected strain N5 (4.2 mg/mL) were comparable, the strain DSM 618 produces three-fold higher amounts (14.9 mg/mL). The largest subtilin yield was obtained from *B. spizizenii* DSM 6405 (33 mg/mL) and the field-collected *B. spizizenii* HS (30 mg/mL). Representative chromatograms for these strains are shown in Fig. 5A. The production yields of the DSM strains 1087, 6395, and 8439 were similar to the ATCC 6633 strain. Our finding that different strains produce different amounts of subtilin––the yield of the HS strain was sevenfold superior to the yield obtained from the original subtilin producer *B. spizizenii* ATCC 6633 (Heinzmann et al. 2006) and imply differential efficiencies in subtilin production. The examined *B. spizizenii* strains may have developed different genetic elements for the regulation of the extremely complex system of subtilin biosynthesis, such as repressor (AbrB) or activator (Sigma factor H) elements or variations in the promoter regions (-35 regions) (Stein et al. 2002b and 2003; Kleerebezem 2004; Kleerebezem et al. 2004; Spieß et al. 2015; Zhang et al, 2022). Furthermore, also the subsequent steps in subtilin biosynthesis for example post-translational dehydration of serine (threonine), addition of neighboring cysteines (Kiesau et al. 1997; Helfrich et al. 2007), and final processing (Stein and Entian 2002; Corvey et al. 2003) might exhibit differential efficiency in the investigated strains.

## GC content of the subtilin gene cluster *spa*

**Fig. 6:**
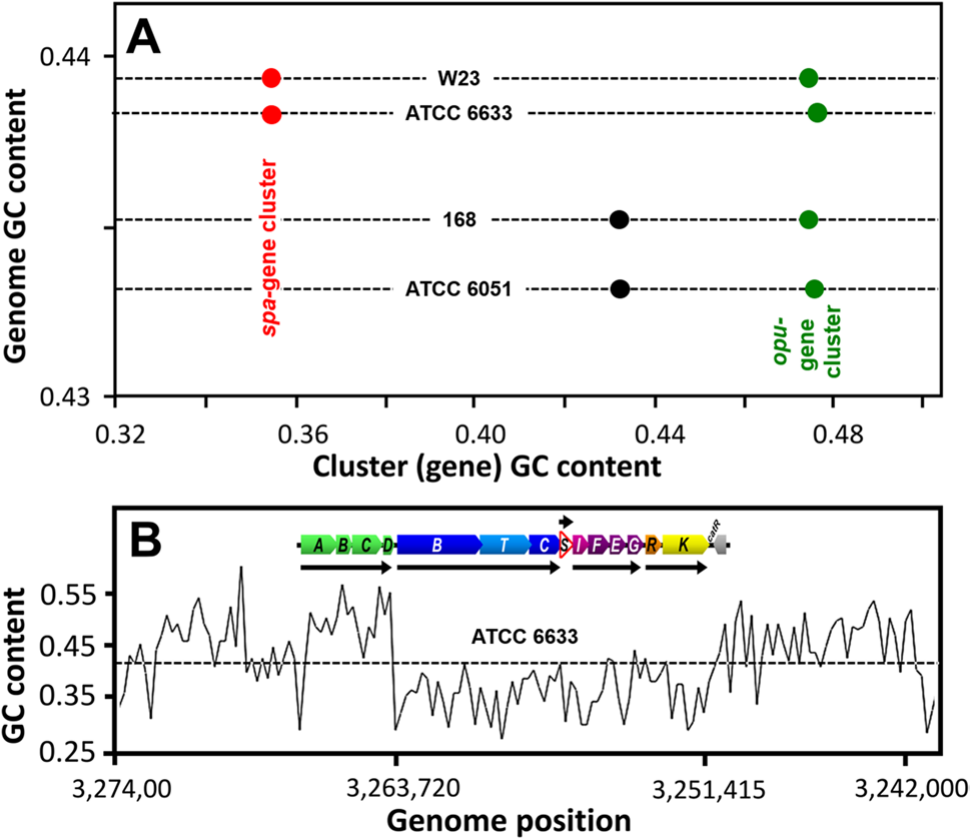
Comparison of the GC content of the subtilin gene cluster and its flanking regions with the GC content of the *B. subtilis* and *B. spizizenii* genomes. **A** The GC content of the 12 kb subtilin gene cluster (red balls) of *B. spizizenii* (W23 and ATCC 6633), the upstream localized *opuB* gene cluster (green), and the downstream localized gene region of subtilin non-producers (black) were plotted versus the GC-content of the cognate genomes (black horizontal dotted lines). B GC content of the *spa* gene cluster of *B. spizizenii* ATCC 6633 and its flanking regions (inverse orientation). Insert: The subtilin cluster of *B. spizizenii* ATCC 6633 encoding the subtilin gene cluster *spaBCSFEGRK* (11.8 kbp, nucleotide positions 3,251,415–3,263,272, negative strand). Red, *spaS* encoding the subtilin pre-propeptide; blue the posttranslational modification machinery *spaBC* and the transporter *spaT*; purple, genes *spaIFEG* involved in immunity against matured subtilin; orange, *spaRK* encoding a two component regulatory system (Stein 2005). Black arrows represent different polycistronic transcripts of the *spa*-cluster. Upstream of the *spa* gene cluster is the *opuB*-operon (green), which is involved in choline transport (Hofmann and Brenner 2017); *catR* downstream of the *spa* cluster belongs to the MarR/DUF24 family of transcription repressors (Chi et al. 2010)

Our results show that for all *B. spizizenii* strains characterized so far, the Sub^+^ phenotype is a characteristic feature. The analysis of the base compositions of a given genome is a common strategy to investigate gene history (Garcia-Vallvé et al. 2000; Popa et al. 2001). Remarkably, in all analyzed *B. spizizenii* genomes, the average GC content of the subtilin gene cluster of 36% is significantly lower (about 8%) than the average GC content of the respective *B. spizizenii* host genome of about 44% (Fig. 6 and Tab. S3, supporting information). This observation is a strong hint that *B. spizizenii* acquired the subtilin gene cluster most likely from another microorganism by a recent horizontal gene transfer event as is hypothesized for a number of lanthipeptide producers (Zhang et al. 2012).

## The subtilin producing ***B. spizizenii*** strains differ from the ***B. spizizenii*** typing strain TU-B-10^ T^ in 16S rDNA and lanthipeptide sequence

Nakamura et al. (1999) proposed the classification of *B. subtilis* strains into two classes: (1) The 168-type strains into *B. subtilis* subsp. *subtilis*, and (2) the W23 strains (Zeigler et al. 2008; Zeigler, 2011) into *B. subtilis* subsp. *spizizenii*. Classical chemotaxonomy differentiates between both classes by the composition of their cell wall teichoic acids, whereas 168-type strains are endowed with the essential major teichoic acids poly(glycerol phosphate) and the non-essential minor teichoic acids poly(glucopyranosyl *N*-acetylgalactosamine 1-phosphate), the W-23-type mainly consists of poly(ribitol phosphate) (Lazarevic et al. 2002). Very recently, *B. subtilis* subsp. *spizizenii* was promoted to species status on the basis of comparative genomics and secondary metabolite (mycosubtilin and bacillaene) production (Dunlap et al. 2020).

The experiments presented in this study have revealed that all *B. spizizenii* strains produced the lanthipeptide subtilin. Surprisingly, a comparison of both, 16S rDNA gene organization and 16S rDNA gene sequences showed that the subtilin producing *B. spizizenii* strains characteristically differ from the *B. spizizenii* typing strain TU-B-10^ T^ (DSM 15029^ T^). *B. spizizenii* ATCC 6633 has ten 16S rRNA encoding genes (*rrn*) identical in length and sequence, whereas only eight *rrn* genes were found for its close relative, the strain W23 (Table S2, Supplement). The *B. spizizenii* typing strain TU-B-10^ T^ contains ten 16S rDNA genes (*rrn* genes) with a different genetic arrangement than the subtilin producing *B. spizizenii* strains: The first five *rrn* genes (*rrn*O, A, J, W, and I) are arranged similarly to the *rrn* gene organization of *B. spizizenii* ATCC 6633 and even *B. subtilis* 168 (Tab. S2 Supplement). However, the *rrn* genes 6–10 are located at different positions in the genome than those of *B. spizizenii* ATCC 6633 and *B. subtilis* 168 indicating genetic restructuring. The 16S rDNA encoding sequence of the subtilin producing *B. spizizenii* strains ATCC 6633, DSM 618, DSM 6405, N5, and HS, have a C, T and C at positions 181, 279, and 600, respectively (Fig. 3A). These three positions can be used to clearly differentiate subtilin producing *B. spizizenii* from subtilin non-producing *B. subtilis* strains (G, C/T, T at positions 181, 279, and 600, respectively). Furthermore, these positions can be even used to differentiate the subtilin producer from the *B. spizizenii* typing strain TU-B-10 T: C, T, T at position 181, 279, and 600, respectively (Fig. 3A). In this context, it is important that for the TU-B-10^ T^ strain, the production of a lanthipeptide (entianin) was described that differs from subtilin in three amino acids: L6V, A15L, and L24I (Fuchs et al. 2011). Taken together, the differences in both the lanthipeptide structures and the organization and sequences of the 16S rRNA-encoding genes suggest the split of *B. spizizenii* into subspecies (Fig. 3B): the entianin producing *B. spizizenii* typing strain TU-B-10^ T^ and the subtilin producing novel *B. spizizenii* subspecies strains.

## Conclusion

In an age in which bacterial resistance to antibiotics is becoming increasingly important, systematic screening for new antibiotic agents with new mechanisms of action is still an important strategy. Microorganisms from the genus *Bacillus* are able to produce a large number of different antimicrobial substances (Stein 2005; Zhao et al. 2018; Tran et al. 2022). It is to be expected that a large number of new active antimicrobial agents and isoforms of known antibiotics with minor chemical modifications will be found in the future through systematic screening, a strategy which is strongly supported by subsequent genome sequencing and novel genome mining bioinformatic tools (Walker et al. 2020). The results of this work show that many *B. spizizenii* strains always produce a cocktail of antibiotic agents (Stein 2004 and 2020; Mülner et al. 2020), in particular several lipophilic membrane-active agents like subtilin, subtilosin, surfactin, and fengycin: In addition to classical 16S rDNA-based typing, determination of individual antibiotic cocktails (e.g., by mass spectrometric analyses) can contribute to very efficient subclassification of *B. subtilis* (Dunlap et al. 2020) and *B. spizizenii* (this study) strains.

## Supplementary Information

Below is the link to the electronic supplementary material.

Supplementary file1 (DOCX 37 KB)
