# Supplementary material for: Antibiotic profiling of wild-type bacilli led to the discovery of new lanthipeptide subtilin-producing Bacillus spizizenii strains whose 16S rDNA sequences differ from the B. spizizenii typing strain
Source: Int Microbiol. 2022 Jul 28;25(4):839–50. doi: 10.1007/s10123-022-00266-5 (PMC9526687; doi:10.1007/s10123-022-00266-5)
Supplement: Supplementary file 1 — Supplementary file1 (DOCX 37 KB) [file 10123_2022_266_MOESM1_ESM.docx]

**Antibiotic profiling of wild-type bacilli led to the discovery of new lanthipeptide subtilin-producing *Bacillus spizizenii* strains whose 16S rDNA sequences differ from the *B.***  ***spizizenii* typing strain**

**Supporting Information**

## Markus Helfrich^2,3^, Karl-Dieter Entian^2^, Torsten Stein^1,2^*

^1^ Chemistry & Molecular Biotechnology, Aalen University, Beethovenstraße 1, D-73430 Aalen, Germany
^2^ Life Sciences, Johann Wolfgang-Goethe-University, Max v. Laue Str. 9, D-60439 Frankfurt/Main, Germany
^3^ Present adress: Jennewein Biotechnologie GmbH, Maarweg 32, D-53619 Rheinbreitbach, Germany

*Correspondence to T. Stein, Chemistry & Mol. Biotechnol.,
Aalen University, Beethovenstraße 1, D-73430 Aalen, Germany.

Phone : (+49) 7361 576-3550
Fax: (+49) 7361 576-2358
Email: [Torsten.Stein@hs-aalen.de](mailto:Torsten.Stein@hs-aalen.de)
**ORCID 0000-0001-5749-8215**

Running title: Subtilin production by *Bacillus spizizenii*

**Table S1: Strain collections**

| **Strain collection** | | **Location** | **Link** |
| --- | --- | --- | --- |
| **ATCC** | American Type Culture Collection | Manassas, VA, USA | <https://www.lgcstandards-atcc.org> |
| **DSM** | Deutsche Stammsammlung für Mikroorganismen | Leibnitz Institute, Braunschweig,  Germany | <https://www.dsmz.de> |
| **IAM/**  **JCM** | Center for Cellular and Molecular Research;  collection transferred to JCM, Japan Collection of Microorganisms | RIKEN BioResearch Resource Center, The University of Tokyo, Japan | <https://jcm.brc.riken.jp/en/> |
| **NCIB** | National Collection of Industrial Bacteria | Aberdeen, Scotland, UK | <https://www.ncimb.com> |
| **NCTC** | National Collection of Type Cultures | Public Health England, Salisbury, UK | <https://www.phe-culturecollections.org.uk/collections/nctc.aspx> |
| **NRS/**  **NRLL** | Northern Regional Research Center; Agricultural Research Service Culture Collection | Peoria, Illinois, USA | <https://nrrl.ncaur.usda.gov> |

**Table S2: Genomic organization of 16S rRNA encoding genes *rrn* in different *Bacillus* strains**

|  | ***B. subtilis*** | | |  | ***B. spizizenii*** | | | |
| --- | --- | --- | --- | --- | --- | --- | --- | --- |
|  | **168** | **ATCC 6051** | |  | **ATCC 6633** | | **W23** | **TU-B-10** |
| Ac.no. | NC_000964.3 | | NZ_CP020102.1 |  | NZ_CP034943.1 | | NC_014479.1 | NC_016047.1 |
| **rrn**^1^ |  | |  | **rrn**^2^ | |  |  |  |
| ***rrnO*** | 9,810 | | 9,850 | **1** | | 9,343 | 9,752 | 9,807 |
| ***rrnA*** | 30,279 | | 30,318 | **2** | | 29,955 | 30,364 | 30,418 |
| ***rrnJ*** | 90,536 | | 90,574 | **3** | | 90,289 | 90,698 | 90,609 |
| ***rrnW*** | 96,392 | | 96,430 | **4** | | 96,212 | - | 96,466 |
| ***rrnI*** | 160,893 | | 160,930 | **5** | | 160,754 | 155,240 | 161,004 |
| ***rrnH*** | 166,500 | | 166,539 | **6** | | 166,367 | 160,853 | 221,481 |
| ***rrnG*** | 171,498 | | 171,541 | **7** | | 171,368 | - | 281,958 |
| ***rrnE*** | 635,433 | | 635,477 | **8** | | 624,744 | 613,686 | 764,374 |
| ***rrnD*** | 946,696 | | 946,736 | **9** | | 917,511 | 906,219 | 1,058,208 |
| ***rrnB*** | 3,177,086 | | 3,177,105 | **10** | | 2,986,967 | 2,963,520 | 3,116,025 |

^1)^ The annotation of the 16S rRNA genes *rrn* of *B. subtilis* 168 is according to Kunst et al. (1997) and was adopted to the highly homologous genes of *B. subtilis* ATCC 6051 (Nye et al. 2017).

^2)^ Temporary annotation of the 16S rRNA genes of *B. spizizenii* ATCC 6633, W23, and TU-B-10^T^ for phylogenetic analyses according to their genomic positions.

**Table S3: GC-content of the subtilin gene cluster (*spa*) in comparison to the GC-content of the corresponding *B. subtilis* and *B. spizizenii* genomes**

|  |  | **(G + C) – content (%)** | | | | |
| --- | --- | --- | --- | --- | --- | --- |
| ***Bacillus* strain^a^** | **Size**  **(mb)** | **genome** | ***opuBD***  **operon**  **(3.2 kb)** | ***spa*-cluster**  **(12.0 kb)** | **downstream**  **region^b^**  **(4.0 kb)** | ***yvaQ^c^***  **(1,7 kb)** |
| ***B. subtilis* 168** | 4.21 | 43.5 | 48.0 | -- | 44.2 | 43.8 |
| ***B. subtilis* ATCC 6051** | 4.30 | 43.3 | 48.1 | -- | 44.2 | 43.8 |
| ***B. spizizenii* ATCC 6633** | 4.05 | 43.8 | 48.2 | 36.0 | 44.3 | -- |
| ***B. spizizenii* W23** | 4.03 | 43.9 | 48.0 | 36.0 | 44.1 | -- |

^a^ NCBI gene bank records for the genomic sequences:
 *B. subtilis* 168: <https://www.ncbi.nlm.nih.gov/nuccore/NC_000964.3> *B. subtilis* ATCC 6051 (NCIB 3610): <https://www.ncbi.nlm.nih.gov/nuccore/NZ_CP020102.1>
 *B. spizizenii* ATCC 6633: <https://www.ncbi.nlm.nih.gov/nuccore/NZ_CP034943.1>
 *B spizizenii* W23: <https://www.ncbi.nlm.nih.gov/nuccore/NC_014479.1>
^b^4 kb gene region downstream of the *spa* gene cluster;
*^c^yvaQ*, a 1.7 kb gene upstream of the *spa* gene cluster is not present in *B. subtilis* 168 and ATCC 6051

**Tab. S4: Multiple Sequence Alignment (MSA) of representative 16S rRNA gene sequences of *B. subtilis* strains (168 and ATCC 6051), and representative *B. spizizenii* strains ATCC 6633 and W23, as well as TU-B-10^T^ (DSM 15029)^a^ using Clustal Omega^b,c^**

168_rrnO atttatcggagagtttgatcctggctcaggacgaacgctggcggcgtgcctaatacatgc

6051_rrnO --ttatcggagagtttgatcctggctcaggacgaacgctggcggcgtgcctaatacatgc

168_rrnJ -tttatcggagagtttgatcctggctcaggacgaacgctggcggcgtgcctaatacatgc

6051_rrnJ --ttatcggagagtttgatcctggctcaggacgaacgctggcggcgtgcctaatacatgc

IP_WT ------------------------------------------------------------

DSM3258 ------------------------------------------------------------

HI_WT ------------------------------------------------------------

6633_6 --ttatcggagagtttgatcctggctcaggacgaacgctggcggcgtgcctaatacatgc

W23_3 --ttatcggagagtttgatcctggctcaggacgaacgctggcggcgtgcctaatacatgc

DSM6405 ------------------------------------------------------------

N5_WT ------------------------------------------------------------

HS_WT ------------------------------------------------------------

DSM_618 ------------------------------------------------------------

TUB10_6 --ttatcggagagtttgatcctggctcaggacgaacgctggcggcgtgcctaatacatgc

168_rrnO aagtcgagcggacagatgggagcttgctccctgatgttagcggcggacgggtgagtaaca

6051_rrnO aagtcgagcggacagatgggagcttgctccctgatgttagcggcggacgggtgagtaaca

168_rrnJ aagtcgagcggacagatgggagcttgctccctgatgttagcggcggacgggtgagtaaca

6051_rrnJ aagtcgagcggacagatgggagcttgctccctgatgttagcggcggacgggtgagtaaca

IP_WT ----------------------cttgctccctgatgttagcggcggacgggtgagtaaca

DSM3258 ----------------------cttgctccctgatgttagcggcggacgggtgagtaaca

HI_WT ----------------------cttgctccctgatgttagcggcggacgggtgagtaaca

6633_6 aagtcgagcggacagatgggagcttgctccctgatgttagcggcggacgggtgagtaaca

W23_3 aagtcgagcggacagatgggagcttgctccctgatgttagcggcggacgggtgagtaaca

DSM6405 ----------------------cttgctccctgatgttagcggcggacgggtgagtaaca

N5_WT ----------------------cttgctccctgatgttagcggcggacgggtgagtaaca

HS_WT ----------------------cttgctccctgatgttagcggcggacgggtgagtaaca

DSM_618 ----------------------cttgctccctgatgttagcggcggacgggtgagtaaca

TUB10_6 aagtcgagcggacagatgggagcttgctccctgatgttagcggcggacgggtgagtaaca

**************************************

168_rrnO cgtgggtaacctgcctgtaagactgggataactccgggaaaccggggctaataccggatg

6051_rrnO cgtgggtaacctgcctgtaagactgggataactccgggaaaccggggctaataccggatg

168_rrnJ cgtgggtaacctgcctgtaagactgggataactccgggaaaccggggctaataccggatg

6051_rrnJ cgtgggtaacctgcctgtaagactgggataactccgggaaaccggggctaataccggatg

IP_WT cgtgggtaacctgcctgtaagactgggataactccgggaaaccggggctaataccggatg

DSM3258 cgtgggtaacctgcctgtaagactgggataactccgggaaaccggggctaataccggatg

HI_WT cgtgggtaacctgcctgtaagactgggataactccgggaaaccggggctaataccggatg

6633_6 cgtgggtaacctgcctgtaagactgggataactccgggaaaccggggctaataccggatg

W23_3 cgtgggtaacctgcctgtaagactgggataactccgggaaaccggggctaataccggatg

DSM6405 cgtgggtaacctgcctgtaagactgggataactccgggaaaccggggctaataccggatg

N5_WT cgtgggtaacctgcctgtaagactgggataactccgggaaaccggggctaataccggatg

HS_WT cgtgggtaacctgcctgtaagactgggataactccgggaaaccggggctaataccggatg

DSM_618 cgtgggtaacctgcctgtaagactgggataactccgggaaaccggggctaataccggatg

TUB10_6 cgtgggtaacctgcctgtaagactgggataactccgggaaaccggggctaataccggatg

************************************************************

**181**

168_rrnO gttgtttgaaccgcatggttcaaacataaaaggtggcttcggctaccacttacagatgga

6051_rrnO gttgtttgaaccgcatggttcaaacataaaaggtggcttcggctaccacttacagatgga

168_rrnJ gttgtttgaaccgcatggttcaaacataaaaggtggcttcggctaccacttgcagatgga

6051_rrnJ gttgtttgaaccgcatggttcaaacataaaaggtggcttcggctaccacttgcagatgga

IP_WT gttgtttgaaccgcatggttcaaacataaaaggtggcttcggctaccacttacagatgga

DSM3258 gttgtttgaaccgcatggttcagacataaaaggtggcttcggctaccacttacagatgga

HI_WT gttgtttgaaccgcatggttcagacataaaaggtggcttcggctaccacttacagatgga

6633_6 cttgtttgaaccgcatggttcaaacataaaaggtggcttcggctaccacttacagatgga

W23_3 cttgtttgaaccgcatggttcaaacataaaaggtggcttcggctaccacttacagatgga

DSM6405 cttgtttgaaccgcatggttcaaacataaaaggtggcttcggctaccacttacagatgga

N5_WT cttgtttgaaccgcatggttcaaacataaaaggtggcttcggctaccacttacagatgga

HS_WT cttgtttgaaccgcatggttcaaacataaaaggtggcttcggctaccacttacagatgga

DSM_618 cttgtttgaaccgcatggttcaaacataaaaggtggcttcggctaccacttacagatgga

TUB10_6 cttgtttgaaccacatggttcaaacataaaaggtggcttcggctaccacttacagatgga

***********.*********.****************************.********

**270**

168_rrnO cccgcggcgcattagctagttggtgaggtaacggctcaccaaggcgacgatgcgtagccg

6051_rrnO cccgcggcgcattagctagttggtgaggtaacggctcaccaaggcgacgatgcgtagccg

168_rrnJ cccgcggcgcattagctagttggtgaggtaatggctcaccaaggcaacgatgcgtagccg

6051_rrnJ cccgcggcgcattagctagttggtgaggtaatggctcaccaaggcaacgatgcgtagccg

IP_WT cccgcggcgcattagctagttggtgaggtaacggctcaccaaggcaacgatgcgtagccg

DSM3258 cccgcggcgcattagctagttggtgaggtaacggctcaccaaggcgacgatgcgtagccg

HI_WT cccgcggcgcattagctagttggtgaggtaacggctcaccaaggcaacgatgcgtagccg

6633_6 cccgcggcgcattagctagttggtgaggtaatggctcaccaaggcaacgatgcgtagccg

W23_3 cccgcggcgcattagctagttggtgaggtaatggctcaccaaggcaacgatgcgtagccg

DSM6405 cccgcggcgcattagctagttggtgaggtaatggctcaccaaggcaacgatgcgtagccg

N5_WT cccgcggcgcattagctagttggtgaggtaatggctcaccaaggcaacgatgcgtagccg

HS_WT cccgcggcgcattagctagttggtgaggtaatggctcaccaaggcaacgatgcgtagccg

DSM_618 cccgcggcgcattagctagttggtgaggtaatggctcaccaaggcaacgatgcgtagccg

TUB10_6 cccgcggcgcattagctagttggtgaggtaatggctcaccaaggcaacgatgcgtagccg

*******************************.*************.**************

168_rrnO acctgagagggtgatcggccacactgggactgagacacggcccagactcctacgggaggc

6051_rrnO acctgagagggtgatcggccacactgggactgagacacggcccagactcctacgggaggc

168_rrnJ acctgagagggtgatcggccacactgggactgagacacggcccagactcctacgggaggc

6051_rrnJ acctgagagggtgatcggccacactgggactgagacacggcccagactcctacgggaggc

IP_WT acctgagagggtgatcggccacactgggactgagacacggcccagactcctacgggaggc

DSM3258 acctgagagggtgatcggccacactgggactgagacacggcccagactcctacgggaggc

HI_WT acctgagagggtgatcggccacactgggactgagacacggcccagactcctacgggaggc

6633_6 acctgagagggtgatcggccacactgggactgagacacggcccagactcctacgggaggc

W23_3 acctgagagggtgatcggccacactgggactgagacacggcccagactcctacgggaggc

DSM6405 acctgagagggtgatcggccacactgggactgagacacggcccagactcctacgggaggc

N5_WT acctgagagggtgatcggccacactgggactgagacacggcccagactcctacgggaggc

HS_WT acctgagagggtgatcggccacactgggactgagacacggcccagactcctacgggaggc

DSM_618 acctgagagggtgatcggccacactgggactgagacacggcccagactcctacgggaggc

TUB10_6 acctgagagggtgatcggccacactgggactgagacacggcccagactcctacgggaggc

************************************************************

168_rrnO agcagtagggaatcttccgcaatggacgaaagtctgacggagcaacgccgcgtgagtgat

6051_rrnO agcagtagggaatcttccgcaatggacgaaagtctgacggagcaacgccgcgtgagtgat

168_rrnJ agcagtagggaatcttccgcaatggacgaaagtctgacggagcaacgccgcgtgagtgat

6051_rrnJ agcagtagggaatcttccgcaatggacgaaagtctgacggagcaacgccgcgtgagtgat

IP_WT agcagtagggaatcttccgcaatggacgaaagtctgacggagcaacgccgcgtgagtgat

DSM3258 agcagtagggaatcttccgcaatggacgaaagtctgacggagcaacgccgcgtgagtgat

HI_WT agcagtagggaatcttccgcaatggacgaaagtctgacggagcaacgccgcgtgagtgat

6633_6 agcagtagggaatcttccgcaatggacgaaagtctgacggagcaacgccgcgtgagtgat

W23_3 agcagtagggaatcttccgcaatggacgaaagtctgacggagcaacgccgcgtgagtgat

DSM6405 agcagtagggaatcttccgcaatggacgaaagtctgacggagcaacgccgcgtgagtgat

N5_WT agcagtagggaatcttccgcaatggacgaaagtctgacggagcaacgccgcgtgagtgat

HS_WT agcagtagggaatcttccgcaatggacgaaagtctgacggagcaacgccgcgtgagtgat

DSM_618 agcagtagggaatcttccgcaatggacgaaagtctgacggagcaacgccgcgtgagtgat

TUB10_6 agcagtagggaatcttccgcaatggacgaaagtctgacggagcaacgccgcgtgagtgat

************************************************************

168_rrnO gaaggttttcggatcgtaaagctctgttgttagggaagaacaagtgccgttcgaataggg

6051_rrnO gaaggttttcggatcgtaaagctctgttgttagggaagaacaagtgccgttcgaataggg

168_rrnJ gaaggttttcggatcgtaaagctctgttgttagggaagaacaagtgccgttcgaataggg

6051_rrnJ gaaggttttcggatcgtaaagctctgttgttagggaagaacaagtgccgttcgaataggg

IP_WT gaaggttttcggatcgtaaagctctgttgttagggaagaacaagtaccgttcgaataggg

DSM3258 gaaggttttcggatcgtaaagctctgttgttagggaagaacaagtgccgttcaaataggg

HI_WT gaaggttttcggatcgtaaagctctgttgttagggaagaacaagtgccgttcaaataggg

6633_6 gaaggttttcggatcgtaaagctctgttgttagggaagaacaagtaccgttcgaataggg

W23_3 gaaggttttcggatcgtaaagctctgttgttagggaagaacaagtaccgttcgaataggg

DSM6405 gaaggttttcggatcgtaaagctctgttgttagggaagaacaagtaccgttcgaataggg

N5_WT gaaggttttcggatcgtaaagctctgttgttagggaagaacaagtaccgttcgaataggg

HS_WT gaaggttttcggatcgtaaagctctgttgttagggaagaacaagtaccgttcgaataggg

DSM_618 gaaggttttcggatcgtaaagctctgttgttagggaagaacaagtaccgttcgaataggg

TUB10_6 gaaggttttcggatcgtaaagctctgttgttagggaagaacaagtaccgttcgaataggg

*********************************************.******.*******

168_rrnO cggtaccttgacggtacctaaccagaaagccacggctaactacgtgccagcagccgcggt

6051_rrnO cggtaccttgacggtacctaaccagaaagccacggctaactacgtgccagcagccgcggt

168_rrnJ cggtaccttgacggtacctaaccagaaagccacggctaactacgtgccagcagccgcggt

6051_rrnJ cggtaccttgacggtacctaaccagaaagccacggctaactacgtgccagcagccgcggt

IP_WT cggtaccttgacggtacctaaccagaaagccacggctaactacgtgccagcagccgcggt

DSM3258 cggcaccttgacggtacctaaccagaaagccacggctaactacgtgccagcagccgcggt

HI_WT cggcaccttgacggtacctaaccagaaagccacggctaactacgtgccagcagccgcggt

6633_6 cggtaccttgacggtacctaaccagaaagccacggctaactacgtgccagcagccgcggt

W23_3 cggtaccttgacggtacctaaccagaaagccacggctaactacgtgccagcagccgcggt

DSM6405 cggtaccttgacggtacctaaccagaaagccacggctaactacgtgccagcagccgcggt

N5_WT cggtaccttgacggtacctaaccagaaagccacggctaactacgtgccagcagccgcggt

HS_WT cggtaccttgacggtacctaaccagaaagccacggctaactacgtgccagcagccgcggt

DSM_618 cggtaccttgacggtacctaaccagaaagccacggctaactacgtgccagcagccgcggt

TUB10_6 cggtaccttgacggtacctaaccagaaagccacggctaactacgtgccagcagccgcggt

***.********************************************************

**600**

168_rrnO aatacgtaggtggcaagcgttgtccggaattattgggcgtaaagggctcgcaggcggttt

6051_rrnO aatacgtaggtggcaagcgttgtccggaattattgggcgtaaagggctcgcaggcggttt

168_rrnJ aatacgtaggtggcaagcgttgtccggaattattgggcgtaaagggctcgcaggcggttt

6051_rrnJ aatacgtaggtggcaagcgttgtccggaattattgggcgtaaagggctcgcaggcggttt

IP_WT aatacgtaggtggcaagcgttgtccggaattattgggcgtaaagggctcgcaggcggttt

DSM3258 aatacgtaggtggcaagcgttgtccggaattattgggcgtaaagggctcgcaggcggttt

HI_WT aatacgtaggtggcaagcgttgtccggaattattgggcgtaaagggctcgcaggcggttt

6633_6 aatacgtaggtggcaagcgttgtccggaattattgggcgtaaagggctcgcaggcggttc

W23_3 aatacgtaggtggcaagcgttgtccggaattattgggcgtaaagggctcgcaggcggttc

DSM6405 aatacgtaggtggcaagcgttgtccggaattattgggcgtaaagggctcgcaggcggttc

N5_WT aatacgtaggtggcaagcgttgtccggaattattgggcgtaaagggctcgcaggcggttc

HS_WT aatacgtaggtggcaagcgttgtccggaattattgggcgtaaagggctcgcaggcggttc

DSM_618 aatacgtaggtggcaagcgttgtccggaattattgggcgtaaagggctcgcaggcggttc

TUB10_6 aatacgtaggtggcaagcgttgtccggaattattgggcgtaaagggctcgcaggcggttt

***********************************************************.

168_rrnO cttaagtctgatgtgaaagcccccggctcaaccggggagggtcattggaaactggggaac

6051_rrnO cttaagtctgatgtgaaagcccccggctcaaccggggagggtcattggaaactggggaac

168_rrnJ cttaagtctgatgtgaaagcccccggctcaaccggggagggtcattggaaactggggaac

6051_rrnJ cttaagtctgatgtgaaagcccccggctcaaccggggagggtcattggaaactggggaac

IP_WT cttaagtctgatgtgaaagcccccggctcaaccggggagggtcattggaaactggggaac

DSM3258 cttaagtctgatgtgaaagcccccggctcaaccggggagggtcattggaaactggggaac

HI_WT cttaagtctgatgtgaaagcccccggctcaaccggggagggtcattggaaactggggaac

6633_6 cttaagtctgatgtgaaagcccccggctcaaccggggagggtcattggaaactggggaac

W23_3 cttaagtctgatgtgaaagcccccggctcaaccggggagggtcattggaaactggggaac

DSM6405 cttaagtctgatgtgaaagcccccggctcaaccggggagggtcattggaaactggggaac

N5_WT cttaagtctgatgtgaaagcccccggctcaaccggggagggtcattggaaactggggaac

HS_WT cttaagtctgatgtgaaagcccccggctcaaccggggagggtcattggaaactggggaac

DSM_618 cttaagtctgatgtgaaagcccccggctcaaccggggagggtcattggaaactggggaac

TUB10_6 cttaagtctgatgtgaaagcccccggctcaaccggggagggtcattggaaactggggaac

************************************************************

168_rrnO ttgagtgcagaagaggagagtggaattccacgtgtagcggtgaaatgcgtagagatgtgg

6051_rrnO ttgagtgcagaagaggagagtggaattccacgtgtagcggtgaaatgcgtagagatgtgg

168_rrnJ ttgagtgcagaagaggagagtggaattccacgtgtagcggtgaaatgcgtagagatgtgg

6051_rrnJ ttgagtgcagaagaggagagtggaattccacgtgtagcggtgaaatgcgtagagatgtgg

IP_WT ttgagtgcagaagaggagagtggaattccacgtgtagcggtgaaatgcgtagagatgtgg

DSM3258 ttgagtgcagaagaggagagtggaattccacgtgtagcggtgaaatgcgtagagatgtgg

HI_WT ttgagtgcagaagaggagagtggaattccacgtgtagcggtgaaatgcgtagagatgtgg

6633_6 ttgagtgcagaagaggagagtggaattccacgtgtagcggtgaaatgcgtagagatgtgg

W23_3 ttgagtgcagaagaggagagtggaattccacgtgtagcggtgaaatgcgtagagatgtgg

DSM6405 ttgagtgcagaagaggagagtggaattccacgtgtagcggtgaaatgcgtagagatgtgg

N5_WT ttgagtgcagaagaggagagtggaattccacgtgtagcggtgaaatgcgtagagatgtgg

HS_WT ttgagtgcagaagaggagagtggaattccacgtgtagcggtgaaatgcgtagagatgtgg

DSM_618 ttgagtgcagaagaggagagtggaattccacgtgtagcggtgaaatgcgtagagatgtgg

TUB10_6 ttgagtgcagaagaggagagtggaattccacgtgtagcggtgaaatgcgtagagatgtgg

************************************************************

168_rrnO aggaacaccagtggcgaaggcgactctctggtctgtaactgacgctgaggagcgaaagcg

6051_rrnO aggaacaccagtggcgaaggcgactctctggtctgtaactgacgctgaggagcgaaagcg

168_rrnJ aggaacaccagtggcgaaggcgactctctggtctgtaactgacgctgaggagcgaaagcg

6051_rrnJ aggaacaccagtggcgaaggcgactctctggtctgtaactgacgctgaggagcgaaagcg

IP_WT aggaacaccagtggcgaaggcgactctctggtctgtaactgacgctgaggagcgaaagcg

DSM3258 aggaacaccagtggcgaaggcgactctctggtctgtaactgacgctgaggagcgaaagcg

HI_WT aggaacaccagtggcgaaggcgactctctggtctgtaactgacgctgaggagcgaaagcg

6633_6 aggaacaccagtggcgaaggcgactctctggtctgtaactgacgctgaggagcgaaagcg

W23_3 aggaacaccagtggcgaaggcgactctctggtctgtaactgacgctgaggagcgaaagcg

DSM6405 aggaacaccagtggcgaaggcgactctctggtctgtaactgacgctgaggagcgaaagcg

N5_WT aggaacaccagtggcgaaggcgactctctggtctgtaactgacgctgaggagcgaaagcg

HS_WT aggaacaccagtggcgaaggcgactctctggtctgtaactgacgctgaggagcgaaagcg

DSM_618 aggaacaccagtggcgaaggcgactctctggtctgtaactgacgctgaggagcgaaagcg

TUB10_6 aggaacaccagtggcgaaggcgactctctggtctgtaactgacgctgaggagcgaaagcg

************************************************************

168_rrnO tggggagcgaacaggattagataccctggtagtccacgccgtaaacgatgagtgctaagt

6051_rrnO tggggagcgaacaggattagataccctggtagtccacgccgtaaacgatgagtgctaagt

168_rrnJ tggggagcgaacaggattagataccctggtagtccacgccgtaaacgatgagtgctaagt

6051_rrnJ tggggagcgaacaggattagataccctggtagtccacgccgtaaacgatgagtgctaagt

IP_WT tggggagcgaacaggattagataccctggtagtccacgccgtaaacgatgagtgctaagt

DSM3258 tggggagcgaacaggattagataccctggtagtccacgccgtaaacgatgagtgctaagt

HI_WT tggggagcgaacaggattagataccctggtagtccacgccgtaaacgatgagtgctaagt

6633_6 tggggagcgaacaggattagataccctggtagtccacgccgtaaacgatgagtgctaagt

W23_3 tggggagcgaacaggattagataccctggtagtccacgccgtaaacgatgagtgctaagt

DSM6405 tggggagcgaacaggattagataccctggtagtccacgccgtaaacgatgagtgctaagt

N5_WT tggggagcgaacaggattagataccctggtagtccacgccgtaaacgatgagtgctaagt

HS_WT tggggagcgaacaggattagataccctggtagtccacgccgtaaacgatgagtgctaagt

DSM_618 tggggagcgaacaggattagataccctggtagtccacgccgtaaacgatgagtgctaagt

TUB10_6 tggggagcgaacaggattagataccctggtagtccacgccgtaaacgatgagtgctaagt

************************************************************

168_rrnO gttagggggtttccgccccttagtgctgcagctaacgcattaagcactccgcctggggag

6051_rrnO gttagggggtttccgccccttagtgctgcagctaacgcattaagcactccgcctggggag

168_rrnJ gttagggggtttccgccccttagtgctgcagctaacgcattaagcactccgcctggggag

6051_rrnJ gttagggggtttccgccccttagtgctgcagctaacgcattaagcactccgcctggggag

IP_WT gttagggggtttccgccccttagtgctgcagctaacgcattaagcactccgcctggggag

DSM3258 gttagggggtttccgccccttagtgctgcagctaacgcattaagcactccgcctggggag

HI_WT gttagggggtttccgccccttagtgctgcagctaacgcattaagcactccgcctggggag

6633_6 gttagggggtttccgccccttagtgctgcagctaacgcattaagcactccgcctggggag

W23_3 gttagggggtttccgccccttagtgctgcagctaacgcattaagcactccgcctggggag

DSM6405 gttagggggtttccgccccttagtgctgcagctaacgcattaagcactccgcctggggag

N5_WT gttagggggtttccgccccttagtgctgcagctaacgcattaagcactccgcctggggag

HS_WT gttagggggtttccgccccttagtgctgcagctaacgcattaagcactccgcctggggag

DSM_618 gttagggggtttccgccccttagtgctgcagctaacgcattaagcactccgcctggggag

TUB10_6 gttagggggtttccgccccttagtgctgcagctaacgcattaagcactccgcctggggag

************************************************************

168_rrnO tacggtcgcaagactgaaactcaaaggaattgacgggggcccgcacaagcggtggagcat

6051_rrnO tacggtcgcaagactgaaactcaaaggaattgacgggggcccgcacaagcggtggagcat

168_rrnJ tacggtcgcaagactgaaactcaaaggaattgacgggggcccgcacaagcggtggagcat

6051_rrnJ tacggtcgcaagactgaaactcaaaggaattgacgggggcccgcacaagcggtggagcat

IP_WT tacggtcgcaagactgaaactcaaaggaattgacgggggcccgcacaagcggtggagcat

DSM3258 tacggtcgcaagactgaaactcaaaggaattgacgggggcccgcacaagcggtggagcat

HI_WT tacggtcgcaagactgaaactcaaaggaattgacgggggcccgcacaagcggtggagcat

6633_6 tacggtcgcaagactgaaactcaaaggaattgacgggggcccgcacaagcggtggagcat

W23_3 tacggtcgcaagactgaaactcaaaggaattgacgggggcccgcacaagcggtggagcat

DSM6405 tacggtcgcaagactgaaactcaaaggaattgacgggggcccgcacaagcggtggagcat

N5_WT tacggtcgcaagactgaaactcaaaggaattgacgggggcccgcacaagcggtggagcat

HS_WT tacggtcgcaagactgaaactcaaaggaattgacgggggcccgcacaagcggtggagcat

DSM_618 tacggtcgcaagactgaaactcaaaggaattgacgggggcccgcacaagcggtggagcat

TUB10_6 tacggtcgcaagactgaaactcaaaggaattgacgggggcccgcacaagcggtggagcat

************************************************************

168_rrnO gtggtttaattcgaagcaacgcgaagaaccttaccaggtcttgacatcctctgacaatcc

6051_rrnO gtggtttaattcgaagcaacgcgaagaaccttaccaggtcttgacatcctctgacaatcc

168_rrnJ gtggtttaattcgaagcaacgcgaagaaccttaccaggtcttgacatcctctgacaatcc

6051_rrnJ gtggtttaattcgaagcaacgcgaagaaccttaccaggtcttgacatcctctgacaatcc

IP_WT gtggtttaattcgaagcaacgcgaagaaccttaccaggtcttgacatcctctgacaatcc

DSM3258 gtggtttaattcgaagcaacgcgaagaaccttaccaggtcttgacatcctctgacaatcc

HI_WT gtggtttaattcgaagcaacgcgaagaaccttaccaggtcttgacatcctctgacaatcc

6633_6 gtggtttaattcgaagcaacgcgaagaaccttaccaggtcttgacatcctctgacaatcc

W23_3 gtggtttaattcgaagcaacgcgaagaaccttaccaggtcttgacatcctctgacaatcc

DSM6405 gtggtttaattcgaagcaacgcgaagaaccttaccaggtcttgacatcctctgacaatcc

N5_WT gtggtttaattcgaagcaacgcgaagaaccttaccaggtcttgacatcctctgacaatcc

HS_WT gtggtttaattcgaagcaacgcgaagaaccttaccaggtcttgacatcctctgacaatcc

DSM_618 gtggtttaattcgaagcaacgcgaagaaccttaccaggtcttgacatcctctgacaatcc

TUB10_6 gtggtttaattcgaagcaacgcgaagaaccttaccaggtcttgacatcctctgacaatcc

************************************************************

168_rrnO tagagataggacgtccccttcgggggcagagtgacaggtggtgcatggttgtcgtcagct

6051_rrnO tagagataggacgtccccttcgggggcagagtgacaggtggtgcatggttgtcgtcagct

168_rrnJ tagagataggacgtccccttcgggggcagagtgacaggtggtgcatggttgtcgtcagct

6051_rrnJ tagagataggacgtccccttcgggggcagagtgacaggtggtgcatggttgtcgtcagct

IP_WT tagagataggacgtccccttcgggggcagagtgacaggtggtgcatggttgtcgtcagct

DSM3258 tagagataggacgtccccttcgggggcagagtgacaggtggtgcatggttgtcgtcagct

HI_WT tagagataggacgtccccttcgggggcagagtgacaggtggtgcatggttgtcgtcagct

6633_6 tagagataggacgtccccttcgggggcagagtgacaggtggtgcatggttgtcgtcagct

W23_3 tagagataggacgtccccttcgggggcagagtgacaggtggtgcatggttgtcgtcagct

DSM6405 tagagataggacgtccccttcgggggcagagtgacaggtggtgcatggttgtcgtcagct

N5_WT tagagataggacgtccccttcgggggcagagtgacaggtggtgcatggttgtcgtcagct

HS_WT tagagataggacgtccccttcgggggcagagtgacaggtggtgcatggttgtcgtcagct

DSM_618 tagagataggacgtccccttcgggggcagagtgacaggtggtgcatggttgtcgtcagct

TUB10_6 tagagataggacgtccccttcgggggcagagtgacaggtggtgcatggttgtcgtcagct

************************************************************

168_rrnO cgtgtcgtgagatgttgggttaagtcccgcaacgagcgcaacccttgatcttagttgcca

6051_rrnO cgtgtcgtgagatgttgggttaagtcccgcaacgagcgcaacccttgatcttagttgcca

168_rrnJ cgtgtcgtgagatgttgggttaagtcccgcaacgagcgcaacccttgatcttagttgcca

6051_rrnJ cgtgtcgtgagatgttgggttaagtcccgcaacgagcgcaacccttgatcttagttgcca

IP_WT cgtgtcgtgagatgttgggttaagtcccgcaacgagcgcaacccttgatcttagttgcca

DSM3258 cgtgtcgtgagatgttgggttaagtcccgcaacgagcgcaacccttgatcttagttgcca

HI_WT cgtgtcgtgagatgttgggttaagtcccgcaacgagcgcaacccttgatcttagttgcca

6633_6 cgtgtcgtgagatgttgggttaagtcccgcaacgagcgcaacccttgatcttagttgcca

W23_3 cgtgtcgtgagatgttgggttaagtcccgcaacgagcgcaacccttgatcttagttgcca

DSM6405 cgtgtcgtgagatgttgggttaagtcccgcaacgagcgcaacccttgatcttagttgcca

N5_WT cgtgtcgtgagatgttgggttaagtcccgcaacgagcgcaacccttgatcttagttgcca

HS_WT cgtgtcgtgagatgttgggttaagtcccgcaacgagcgcaacccttgatcttagttgcca

DSM_618 cgtgtcgtgagatgttgggttaagtcccgcaacgagcgcaacccttgatcttagttgcca

TUB10_6 cgtgtcgtgagatgttgggttaagtcccgcaacgagcgcaacccttgatcttagttgcca

************************************************************

168_rrnO gcattcagttgggcactctaaggtgactgccggtgacaaaccggaggaaggtggggatga

6051_rrnO gcattcagttgggcactctaaggtgactgccggtgacaaaccggaggaaggtggggatga

168_rrnJ gcattcagttgggcactctaaggtgactgccggtgacaaaccggaggaaggtggggatga

6051_rrnJ gcattcagttgggcactctaaggtgactgccggtgacaaaccggaggaaggtggggatga

IP_WT gcattcagttgggcactctaaggtgactgccggtgacaaaccggaggaaggtggggatga

DSM3258 gcattcagttgggcactctaaggtgactgccggtgacaaaccggaggaaggtggggatga

HI_WT gcattcagttgggcactctaaggtgactgccggtgacaaaccggaggaaggtggggatga

6633_6 gcattcagttgggcactctaaggtgactgccggtgacaaaccggaggaaggtggggatga

W23_3 gcattcagttgggcactctaaggtgactgccggtgacaaaccggaggaaggtggggatga

DSM6405 gcattcagttgggcactctaaggtgactgccggtgacaaaccggaggaaggtggggatga

N5_WT gcattcagttgggcactctaaggtgactgccggtgacaaaccggaggaaggtggggatga

HS_WT gcattcagttgggcactctaaggtgactgccggtgacaaaccggaggaaggtggggatga

DSM_618 gcattcagttgggcactctaaggtgactgccggtgacaaaccggaggaaggtggggatga

TUB10_6 gcattcagttgggcactctaaggtgactgccggtgacaaaccggaggaaggtggggatga

************************************************************

168_rrnO cgtcaaatcatcatgccccttatgacctgggctacacacgtgctacaatggacagaacaa

6051_rrnO cgtcaaatcatcatgccccttatgacctgggctacacacgtgctacaatggacagaacaa

168_rrnJ cgtcaaatcatcatgccccttatgacctgggctacacacgtgctacaatggacagaacaa

6051_rrnJ cgtcaaatcatcatgccccttatgacctgggctacacacgtgctacaatggacagaacaa

IP_WT cgtcaaatcatcatgccccttatgacctgggctacacacgtgctacaatggacagaacaa

DSM3258 cgtcaaatcatcatgccccttatgacctgggctacacacgtgctacaatggacagaacaa

HI_WT cgtcaaatcatcatgccccttatgacctgggctacacacgtgctacaatggacagaacaa

6633_6 cgtcaaatcatcatgccccttatgacctgggctacacacgtgctacaatggacagaacaa

W23_3 cgtcaaatcatcatgccccttatgacctgggctacacacgtgctacaatggacagaacaa

DSM6405 cgtcaaatcatcatgccccttatgacctgggctacacacgtgctacaatggacagaacaa

N5_WT cgtcaaatcatcatgccccttatgacctgggctacacacgtgctacaatggacagaacaa

HS_WT cgtcaaatcatcatgccccttatgacctgggctacacacgtgctacaatggacagaacaa

DSM_618 cgtcaaatcatcatgccccttatgacctgggctacacacgtgctacaatggacagaacaa

TUB10_6 cgtcaaatcatcatgccccttatgacctgggctacacacgtgctacaatggacagaacaa

************************************************************

168_rrnO agggcagcgaaaccgcgaggttaagccaatcccacaaatctgttctcagttcggatcgca

6051_rrnO agggcagcgaaaccgcgaggttaagccaatcccacaaatctgttctcagttcggatcgca

168_rrnJ agggcagcgaaaccgcgaggttaagccaatcccacaaatctgttctcagttcggatcgca

6051_rrnJ agggcagcgaaaccgcgaggttaagccaatcccacaaatctgttctcagttcggatcgca

IP_WT agggcagcgaaaccgcgaggttaagccaatcccacaaatctgttctcagttcggatcgca

DSM3258 agggcagcgaaaccgcgaggttaagccaatcccacaaatctgttctcagttcggatcgca

HI_WT agggcagcgaaaccgcgaggttaagccaatcccacaaatctgttctcagttcggatcgca

6633_6 agggcagcgaaaccgcgaggttaagccaatcccacaaatctgttctcagttcggatcgca

W23_3 agggcagcgaaaccgcgaggttaagccaatcccacaaatctgttctcagttcggatcgca

DSM6405 agggcagcgaaaccgcgaggttaagccaatcccacaaatctgttctcagttcggatcgca

N5_WT agggcagcgaaaccgcgaggttaagccaatcccacaaatctgttctcagttcggatcgca

HS_WT agggcagcgaaaccgcgaggttaagccaatcccacaaatctgttctcagttcggatcgca

DSM_618 agggcagcgaaaccgcgaggttaagccaatcccacaaatctgttctcagttcggatcgca

TUB10_6 agggcagcgaaaccgcgaggttaagccaatcccacaaatctgttctcagttcggatcgca

************************************************************

168_rrnO gtctgcaactcgactgcgtgaagctggaatcgctagtaatcgcggatcagcatgccgcgg

6051_rrnO gtctgcaactcgactgcgtgaagctggaatcgctagtaatcgcggatcagcatgccgcgg

168_rrnJ gtctgcaactcgactgcgtgaagctggaatcgctagtaatcgcggatcagcatgccgcgg

6051_rrnJ gtctgcaactcgactgcgtgaagctggaatcgctagtaatcgcggatcagcatgccgcgg

IP_WT gtctgcaactcgactgcgtgaagctggaatcgctagtaatcgcggatcagcatgccgcgg

DSM3258 gtctgcaactcgactgcgtgaagctggaatcgctagtaatcgcggatcagcatgccgcgg

HI_WT gtctgcaactcgactgcgtgaagctggaatcgctagtaatcgcggatcagcatgccgcgg

6633_6 gtctgcaactcgactgcgtgaagctggaatcgctagtaatcgcggatcagcatgccgcgg

W23_3 gtctgcaactcgactgcgtgaagctggaatcgctagtaatcgcggatcagcatgccgcgg

DSM6405 gtctgcaactcgactgcgtgaagctggaatcgctagtaatcgcggatcagcatgccgcgg

N5_WT gtctgcaactcgactgcgtgaagctggaatcgctagtaatcgcggatcagcatgccgcgg

HS_WT gtctgcaactcgactgcgtgaagctggaatcgctagtaatcgcggatcagcatgccgcgg

DSM_618 gtctgcaactcgactgcgtgaagctggaatcgctagtaatcgcggatcagcatgccgcgg

TUB10_6 gtctgcaactcgactgcgtgaagctggaatcgctagtaatcgcggatcagcatgccgcgg

************************************************************

168_rrnO tgaatacgttcccgggccttgtacacaccgcccgtcacaccacgagagtttgtaacaccc

6051_rrnO tgaatacgttcccgggccttgtacacaccgcccgtcacaccacgagagtttgtaacaccc

168_rrnJ tgaatacgttcccgggccttgtacacaccgcccgtcacaccacgagagtttgtaacaccc

6051_rrnJ tgaatacgttcccgggccttgtacacaccgcccgtcacaccacgagagtttgtaacaccc

IP_WT tgaatacgttcccgggccttgtacacaccgcccgtcacaccacgagagtttgtaacaccc

DSM3258 tgaatacgttcccgggccttgtacacaccgcccgtcacaccacgagagtttgtaacaccc

HI_WT tgaatacgttcccgggccttgtacacaccgcccgtcacaccacgagagtttgtaacaccc

6633_6 tgaatacgttcccgggccttgtacacaccgcccgtcacaccacgagagtttgtaacaccc

W23_3 tgaatacgttcccgggccttgtacacaccgcccgtcacaccacgagagtttgtaacaccc

DSM6405 tgaatacgttcccgggccttgtacacaccgcccgtcacaccacgagagtttgtaacaccc

N5_WT tgaatacgttcccgggccttgtacacaccgcccgtcacaccacgagagtttgtaacaccc

HS_WT tgaatacgttcccgggccttgtacacaccgcccgtcacaccacgagagtttgtaacaccc

DSM_618 tgaatacgttcccgggccttgtacacaccgcccgtcacaccacgagagtttgtaacaccc

TUB10_6 tgaatacgttcccgggccttgtacacaccgcccgtcacaccacgagagtttgtaacaccc

************************************************************

168_rrnO gaagtcggtgaggtaaccttttaggagccagccgccgaaggtgggacagatgattggggt

6051_rrnO gaagtcggtgaggtaaccttttaggagccagccgccgaaggtgggacagatgattggggt

168_rrnJ gaagtcggtgaggtaaccttttaggagccagccgccgaaggtgggacagatgattggggt

6051_rrnJ gaagtcggtgaggtaaccttttaggagccagccgccgaaggtgggacagatgattggggt

IP_WT gaagtcggtgaggtaaccttt---------------------------------------

DSM3258 gaagtcggtgaggtaaccttt---------------------------------------

HI_WT gaagtcggtgaggtaaccttt---------------------------------------

6633_6 gaagtcggtgaggtaaccttttaggagccagccgccgaaggtgggacagatgattggggt

W23_3 gaagtcggtgaggtaaccttttaggagccagccgccgaaggtgggacagatgattggggt

DSM6405 gaagtcggtgaggtaaccttt---------------------------------------

N5_WT gaagtcggtgaggtaaccttt---------------------------------------

HS_WT gaagtcggtgaggtaaccttt---------------------------------------

DSM_618 gaagtcggtgaggtaaccttt---------------------------------------

TUB10_6 gaagtcggtgaggtaaccttttaggagccagccgccgaaggtgggacagatgattggggt

*********************

168_rrnO gaagtcgtaacaaggtagccgtatcggaaggtgcggctggatcacctcctttcta

6051_rrnO gaagtcgtaacaaggtagccgtatcggaaggtgcggctggatcacctcctttcta

168_rrnJ gaagtcgtaacaaggtagccgtatcggaaggtgcggctggatcacctcctttcta

6051_rrnJ gaagtcgtaacaaggtagccgtatcggaaggtgcggctggatcacctccttt---

IP_WT -------------------------------------------------------

DSM3258 -------------------------------------------------------

HI_WT -------------------------------------------------------

6633_6 gaagtcgtaacaaggtagccgtatcggaaggtgcggctggatcacctcctttcta

W23_3 gaagtcgtaacaaggtagccgtatcggaaggtgcggctggatcacctcctttcta

DSM6405 -------------------------------------------------------

N5_WT -------------------------------------------------------

HS_WT -------------------------------------------------------

DSM_618 -------------------------------------------------------

TUB10_6 gaagtcgtaacaaggtagccgtatcggaaggtgcggctggatcacctcctttcta

^a^The 16S rRNA genes of *B. subtilis* strains DSM 3258, DSM 6405, and DSM 618, as well as the field collected *Bacillus* strains HI, IP, N5, and HS were PCR amplified and sequenced (accession number: [NCBI GeneBank-link](https://www.ncbi.nlm.nih.gov/nuccore/))
 DSM 6405: DQ452508: <https://www.ncbi.nlm.nih.gov/nuccore/DQ452508>
 DSM 3258: DQ452509: <https://www.ncbi.nlm.nih.gov/nuccore/DQ452509>
 DSM 618: DQ529249: <https://www.ncbi.nlm.nih.gov/nuccore/DQ529249>
 N5: DQ452510: <https://www.ncbi.nlm.nih.gov/nuccore/DQ452510>
 HS1: DQ452511: <https://www.ncbi.nlm.nih.gov/nuccore/DQ452511>
 IP: DQ452512: <https://www.ncbi.nlm.nih.gov/nuccore/DQ452512>
 HI: DQ452513: <https://www.ncbi.nlm.nih.gov/nuccore/DQ452513>
and aligned to 16S rRNA genes of
 *B. subtilis* 168: <https://www.ncbi.nlm.nih.gov/nuccore/NC_000964.3>,
 *B spizizenii* W23: <https://www.ncbi.nlm.nih.gov/nuccore/NC_014479.1>, and
 *B. spizizenii* TU-B-10^T^: <https://www.ncbi.nlm.nih.gov/nuccore/NC_016047.1>
^b^ Clustal Omega 1.2.4: <https://www.ncbi.nlm.nih.gov/nuccore/NC_016047.1> (Sievers et al. 2011)
^c^ Indicator positions for species/subspecies differentiation 181, 270, and 600 are highlighted in red.

**References (Supporting information)**

Kunst F, Ogasawara N, Moszer I et al (1997) The complete genome sequence of the Gram-positive bacterium *Bacillus subtilis.* Nature Nov 390:249-256. <https://doi.org/10.1038/36786>

Nye TM, Schroeder JW, Kearns DB, Simmons LA (2017) Complete Genome Sequence of Undomesticated *Bacillus subtilis* Strain NCIB 3610. Genome Announc 18;5:e00364-17. <https://mra.asm.org/content/5/20/e00364-17>

Sievers F, Wilm A, Dineen D, Gibson TJ, Karplus K, Li W, Lopez R, McWilliam H, Remmert M, Söding J, Thompson JD, Higgins DG (2011) **Fast, scalable generation of high-quality protein multiple sequence alignments using Clustal Omega.** Mol Syst Biol 7:539. <https://doi.org/10.1038/msb.2011.75>
